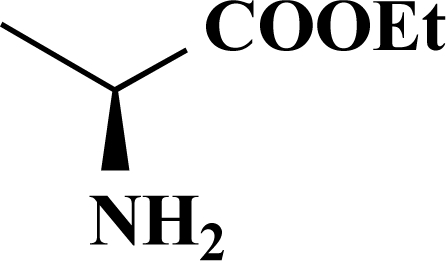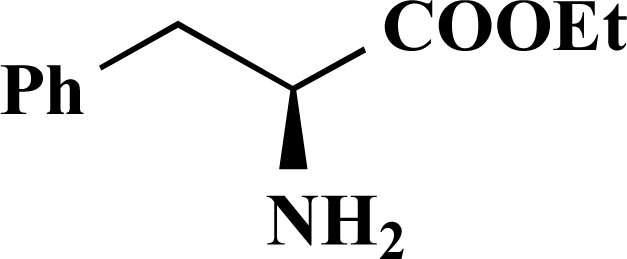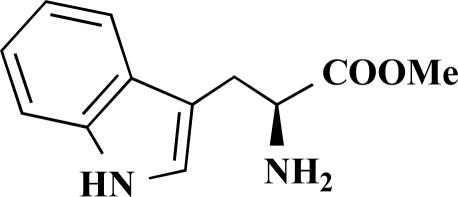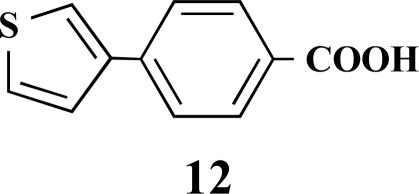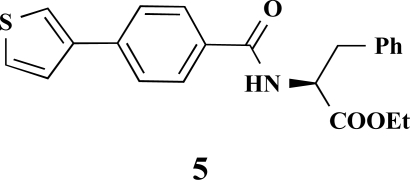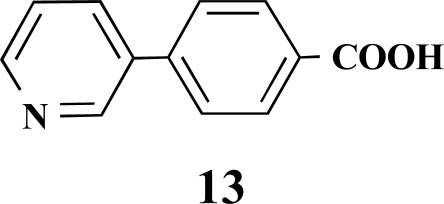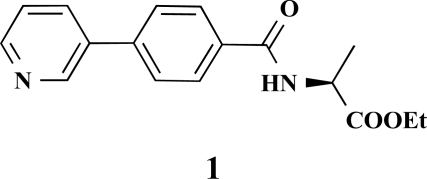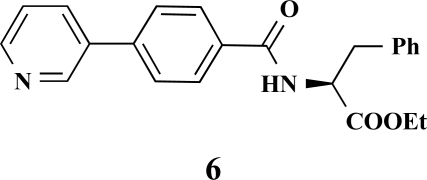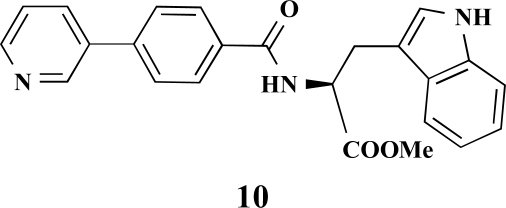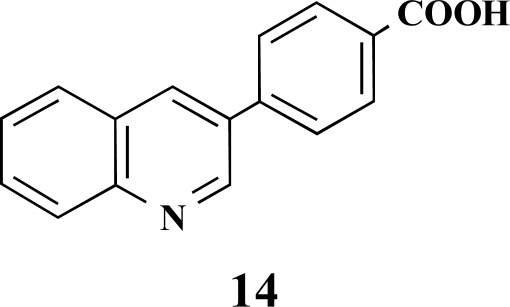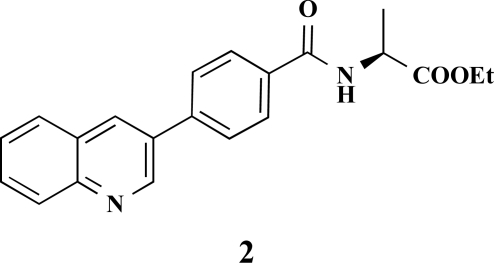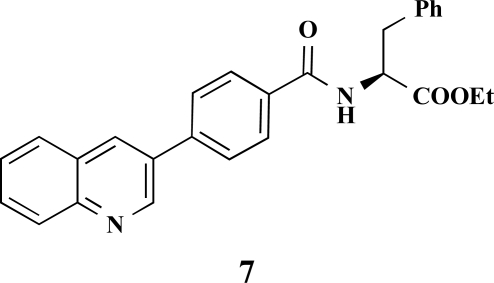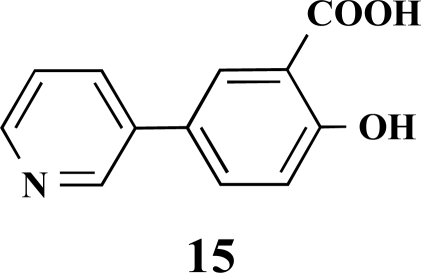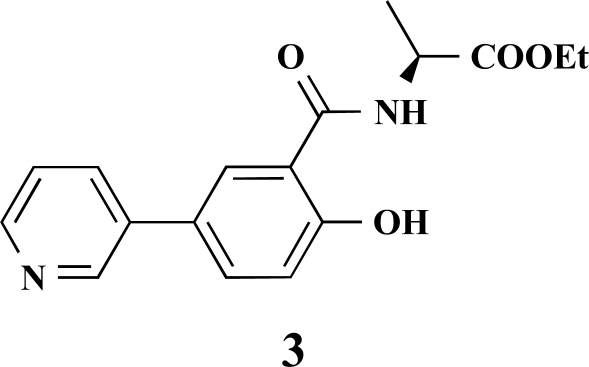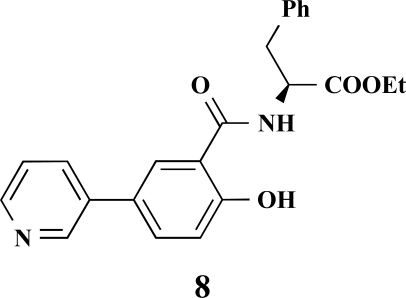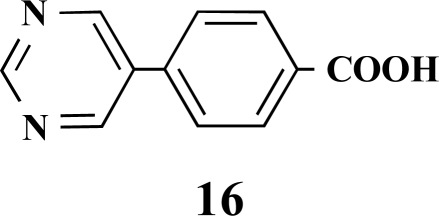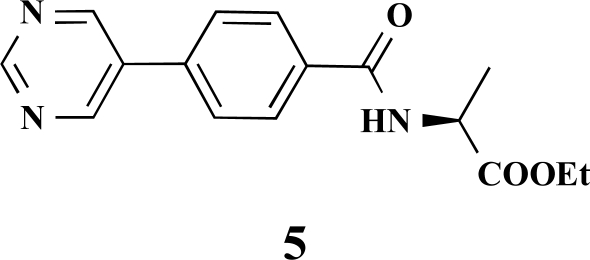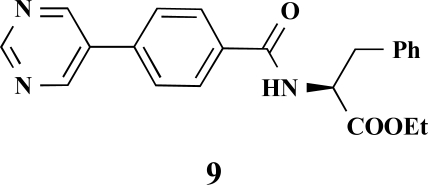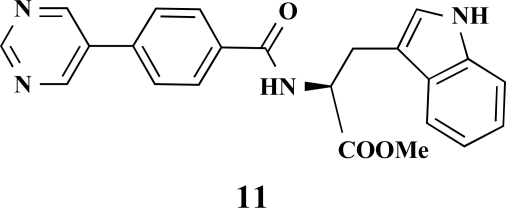# Microwave-Assisted Syntheses of Amino Acid Ester Substituted Benzoic Acid Amides: Potential Inhibitors of Human CD81-Receptor HCV-E2 Interaction

**DOI:** 10.2174/1874104500802010021

**Published:** 2008-04-15

**Authors:** Marcel Holzer, Sigrid Ziegler, Bernd Kronenberger, Christian D Klein, Rolf W Hartmann

**Affiliations:** Pharmaceutical and Medicinal Chemistry, Saarland University, PO Box 151150, D-66041 Saarbrücken, Germany

**Keywords:** Hepatitis C virus, microwave assisted amide syntheses, CD81-receptor, large extracellular loop, benzyl salicylate, protein-protein interaction.

## Abstract

Results from our group showed benzyl salicylate to be a moderate inhibitor of the CD81-LEL–HCV-E2 interaction. To increase the biological activity, heterocyclic substituted benzoic acids were coupled to amino acid esters *via *microwave assisted DCC-reaction. The prepared compounds were tested for their inhibitory potency by means of a fluorescence labeled antibody assay system using HUH7.5 cells.

## 1 INTRODUCTION

Recently the World Health Organization (WHO) estimated that 3% of the world’s population has been infected with the Hepatitis C Virus (HCV) [1]. Infection with HCV is the most common cause of chronic hepatitis with frequent progression to liver cirrhosis and its sequelae [2]. Inhibition of the Hepatitis C Virus E2 glycoprotein (HCV-E2) binding to the *large* *extracellular loop *(LEL) of the human cell surface protein CD81, a member of the tetraspanin family, prevents infection of human hepatocytes, the HCV principal target cells [3]. The aim of the present work was to prepare compounds which bind to CD81-LEL and therefore inhibit the CD81-LEL–HCV-E2 interaction.

**Fig. (1):**
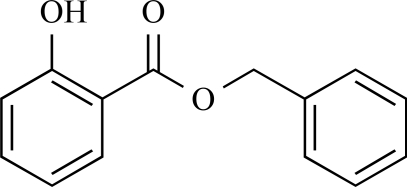
Benzyl salicylate.

This work was based on results recently achieved in our group [4]. Briefly, we found benzyl salicylate (Fig. **1**) to inhibit the CD81-LEL–HCV-E2 interaction (25% at 50 µM) as outcome of a virtual screening followed by biological testing. Several heterocy-clic-substituted benzoic acid amides were synthesized and tested for their biological activity. Some of the prepared compounds showed inhibition of the CD81-LEL–HCV-E2 interaction.

## 2 RESULTS AND DISCUSSION

### 2.1 Structure Modification

Based on the docking pose of benzyl salicylate into the published x-ray structure of CD81-LEL (pdb code: 1IV5) [5], we assumed that an extension of the planar/aromatic ring system could increase the affinity to our intended binding site within a superficial cleft formed by two alpha-helices in the CD81-LEL structure. In order to maximize more specific hydrogen-bonding and electrostatic interactions with the target protein, we decided to introduce heterocyclic ring systems and amino acid moieties in opposite positions of an aromatic core structure. The aim of this work was therefore to prepare compounds of the general structure given in Fig. (**2**) and to determine their inhibition of the CD81-LEL–HCV-E2 interaction.

### 2.2 Syntheses and Biological Testing

**Scheme 1:**
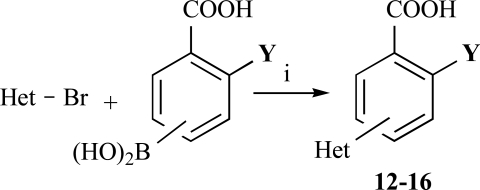
Reagents and conditions: (i) 10 ml EtOH + 15 ml 10% Na_2_CO_3_-solution, Pd(PPh_3_)_4_, O_2_-free, 90ºC over night (Y = -H, -OH).

In the first step of the preparation, starting from commercially available reagents, the bromo substituted aromatic heterocycles were coupled to the corresponding boronic acids with tetrakis(triphenylphosphine) palladium (0) as catalyst via a Suzuki coupling reaction (cf. Scheme **1**) in satisfying yields. The synthesized heterocyclic substituted carboxylic acids **12-16** as well as the applied bromo substituted heterocycles and boronic acids are shown in Table **1**.

**Scheme 2:**
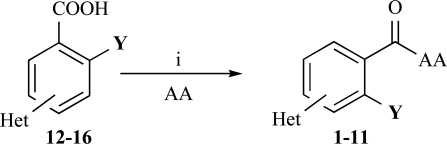
Reagents and conditions: (i) Dicyclohexyl carbodiimide, dimethoxyethane / dichloromethane (5/1), microwaves: 225 Watt, 135ºC, 5 bar, 10 minutes. Het = Heterocycle, AA = amino acid ester: L-alanine ethyl ester, L-phenylalanine ethyl ester, L-tryptophane methyl ester, Y = -H, -OH.

The desired amides **1-11** (Table **2**) were obtained in the second step by connecting the benzoic acids **12-16** to L-alanine ethyl ester, L-phenylalanine ethyl ester and L-tryptophane methyl ester using N,N’-dicyclohexyl carbodiimide (DCC) as coupling agent for microwave-assisted amide formation (cf. Scheme **2**).

Before we decided to apply microwave-assisted amide syntheses, activation of the carboxylic acids by means of thionyl chloride followed by addition of the corresponding amino acid esters was tried. This did not lead to the desired compounds.

DCC coupling reaction under standard conditions was performed next leading to the desired amides in very poor yields. Therefore microwave-assisted DCC coupling reaction was tried to increase the yield of the desired products. This attempt finally led to the target compounds **1-11** in satisfying yields.

A medium throughput assay developed in our group [6] was used to test the prepared compounds **1-11** for their biological activity. This assay is based on the procedure of Pileri *et al. * [7] in which the inhibition of the interaction of the fluorescence-labeled CD81 antibody JS81 with HUH7.5 cells caused by our compounds is determined by FACS. The synthesized compounds **1-11** showed no increased inhibition concerning the CD81-LEL–HCV-E2 interaction compared to the original hit compound benzyl salicylate.

## 3 EXPERIMENTAL SECTION

### 3.1 General Procedure for the Suzuki Coupling Reaction

The boronic acid (1 equivalent) and the bromo substituted heterocycle (1 equivalent) were added to a mixture of 10 mL ethanol and 15 mL sodium carbonate solution (10%). This solution was free from oxygen by evacuating and flushing with nitrogen several times. After addition of 4 mol% of tetrakis(triphenylphosphine)palladium (0) the mixture was stirred at 90°C over night.

The remaining solid was filtered off at that temperature. Subsequently half of the solvent was removed under vacuum. The product precipitated after acidifying to pH 2 using formic acid. It was filtered off and dried under high vacuum.

### 3.2 General Procedure for the Formation of the Amides

The carboxylic acid was stirred with an equivalent amount of the amino acid ester and dicyclohexyl carbodiimide (1.2 equivalent) in the microwave oven (225 Watt, 135°C, 5 bar, 10 minutes) using dry dichloromethane (4 mL) as solvent. After filtration the solvent was removed. Purification of the crude product was performed with column chromatography.

### 3.3 Biological Test

HUH7.5 cells (1*10^5^) were incubated with 100 µl of the potential inhibitor (50 µM + 1% DMSO) in 96 transwell plates for 10 minutes at room temperature. Next 4 µL of the fluorescence-labeled CD81 antibody JS81 and 21 µL of phosphate buffered saline buffer (PBS) were added and kept at room temperature for 10 minutes. After appending 125 µL of PBS the cell suspension was incubated in the dark for 5 hours followed by FACS analysis.

### 3.4 Chemistry

Solvents and reagents were used as received from commercial distributors without further purification. Anhydrous reactions were conducted under a nitrogen atmosphere. Proton and carbon NMR spectra were recorded at a Bruker AM 500. The proton NMR spectra were recorded at 500 MHz, the carbon NMR spectra at 125 MHz. Chemical shifts δ are reported in ppm units. Molecular mass was determined by liquid chromatography – tandem mass spectrometry (LC-MS/MS) using a TSQ Quantum from Thermo Finnigan equipped with an electro spray interface and connected to a Surveyor HPLC (Thermo Finnigan). Positive and negative ion mass spectra were recorded (mass range m/z 150–1500) in normal scan mode. Melting points were determined using a Stuart Scientific SMP3 melting point apparatus. IR measurements were performed on a Bruker Vector 33 at a frequency range from 4000-250 cm^-1^. Wave numbers υ are reported in cm^-1^. Flash chromatography using Merck silica gel 35/40–63/70. Microwave assisted syntheses was performed using a CEM DISCOVER microwave oven.

**(*S*)-Ethyl 2-[4-(pyridin-3-yl)benzamide]propanoate (1). **Yield 24%. mp 118 °C IR 3330, 2930, 2851, 2478, 1745, 1606, 1479, 1437, 1210, 1170 ^1^H-NMR (CDCl_3_) 8.75–8.74 (1 H, m), 8.52–51 (1 H, m), 7.90–7.88 (3 H, m), 7.60 (1 H, d, *J* = 8.51), 7.40–7.37 (1 H, m), 7.29–7.27 (1 H, m), 4.72–4.70 (1 H, m), 4.21–4.17 (2 H, q, *J* = 7.25), 1.48 (3 H, d, *J* = 7.25), 1.24 (3 H, t, *J* = 7.25) ^13^C-NMR (CDCl_3_) 173.38, 166.86, 148.53, 147.70, 140.75, 135.86, 134.97, 127.97, 127.20, 123.95, 61.70, 33.70, 18.13, 14.00 LC/MS-MS 299.11 (M+H^+^).

**(*S*)-Ethyl 2-[4-(quinolin-3-yl)benzamido]propano-ate (2).** Yield 29%. mp 142 °C IR 3356, 2985, 1742, 1638, 1532, 1439, 1205, 1172 ^1^H-NMR (CDCl_3_) 9.35 (1 H, s), 8.81 (1 H, s), 8.25–8.20 (4 H, m), 8.14–8.09 (3 H, m), 7.99–7.94 (1 H, m), 7.85–7.80 (1 H, m), 4.84–4.77 (1 H, m), 4.42–4.38 (2 H, m), 1.72–1.69 (3 H, m), 1.47 (3 H, t, *J* = 7.25) ^13^C-NMR (CDCl_3_) 174.42, 169.59, 150.22, 148.12, 141.95, 135.74, 134.85, 131.46, 129.72, 129.59, 129.54, 129.27, 128.99, 128.68, 128.45, 128.21, 62.41, 50.35, 17.28, 14.50 LC/MS-MS 349.05 (M+H^+^).

**(*S*)-Ethyl 2-[4-hydroxy-3-(pyridin-3-yl)benzamide] propanoate (3). **Yield 14%. IR 2926, 1738, 1642, 1541, 1472, 1376, 1294, 1205 ^1^H-NMR (CDCl_3_) 9.01 (1 H, s), 8.67–8.66 (1 H, m), 8.40–8.39 (1 H, m), 8.29–8.27 (1 H, m), 7.91 (1 H, dd, *J* = 8.51), 7.84–7.80 (1 H, m), 7.70–7.67 (1 H, m), 7.24 (1 H, d, *J* = 8.51), 4.85–4.81 (1 H, m), 4.41–4.39 (2 H, m), 1.71 (3 H, d, *J* = 7.25), 1.45 (3 H, t, *J* = 7.25) ^13^C-NMR (CDCl_3_) 174.31, 170.15, 161.51, 148.31, 147.98, 136.00, 133.44, 133.05, 129.48, 128.15, 125.43, 119.49, 117.49, 62.49, 50.02, 17.48, 14.47 LC/MS-MS 314.97 (M+H^+^).

**(*S*)-Ethyl 2-[4-(pyrimidin-5-yl)benzamido]propanoate (4). **Yield 44%. mp 124 °C IR 2930, 2475, 1741, 1625, 1550, 1415, 1213, 1161 ^1^H-NMR (d_4_-MeOH) 9.35 (1 H, s), 9.30 (2 H, s), 8.22 (2 H, d, *J* = 8.55), 8.02 (2 H, d, *J* = 8.56), 4.82–4.78 (1 H, m), 4.42–4.37 (2 H, m), 1.71 (3 H, d, *J* = 7.57), 1.46 (3 H, t, *J* = 7.25) ^13^C-NMR (d_4_-MeOH) 174.35, 169.36, 158.47, 156.30, 138.54, 135.71, 135.06, 129.66, 128.28, 62.41, 50.34, 17.25, 14.50 LC/MS-MS 300.02 (M+H^+^).

**(*S*)-Ethyl 3-phenyl-2-[4-(thiophen-3-yl)benzamido] propanoate (5). **Yield 43%. mp 160 °C IR 3345, 2997, 1748, 1634, 1517, 1178 ^1^H-NMR (CDCl_3_) 7.76 (2 H, d, *J* = 8.51), 7.64 (2 H, d, *J* = 8.51), 7.54–7.53 (1 H, m), 7.42–7.41 (2 H, m), 7.31–7.25 (3 H, m), 7.17–7.15 (2 H, m), 5.11–5.07 (1 H, m), 4.23 (2 H, q, *J* = 6.94), 3.32–3.23 (2 H, m), 1.28 (3 H, t, *J* = 7.25) ^13^C-NMR (CDCl_3_) 171.66, 166.38, 141.16, 139.07, 135.94, 132.32, 129.45, 128.58, 127.65, 127.14, 126.67, 126.47, 126.16, 121.61, 61.67, 53.56, 37.99, 14.17 LC/MS-MS 380.00 (M+H^+^).

**(*S*)-Ethyl 3-phenyl-2-[4-(pyridin-3-yl)benzamido] propanoate (6). **Yield 11%. mp 170 °C IR 3331, 2929, 1735, 1628, 1537, 1311, 1197 ^1^H-NMR (CDCl_3_) 8.86 (1 H, s), 8.64–8.63 (1 H, m), 7.90–7.88 (1 H, m), 7.84 (2 H, d, *J* = 8.51), 7.64 (2 H, d, *J* = 8.51), 7.40–7.39 (1 H, m), 7.32–7.30 (2 H, m), 7.19–7.15 (3 H, m), 6.66–6.64 (1 H, m), 5.30 (2 H, s), 5.11–5.07 (1 H, m), 4.25–4.21 (2 H, m), 1.31–1.28 (3 H, m) LC/MS-MS 375.10 (M+H^+^).

**(*S*)-Ethyl 3-phenyl-2-[4-(quinolin-3-yl)benzamido] propanoate (7). **Yield 35%. mp 77 °C IR 3324, 2929, 2851, 1741, 1626, 1572, 1529, 1208 ^1^H-NMR (CDCl_3_) 9.18 (1 H, s), 8.34–8.33 (1 H, m), 8.16–8.14 (1 H, d, *J* = 8.51), 7.91–7.88 (3 H, m), 7.79–7.73 (3 H, m), 7.61–7.59 (1 H, m), 7.32–7.28 (3 H, m), 7.18–7.17 (2 H, m), 6.71–6.69 (1 H, m), 5.12–5.09 (1 H, m), 4.25 (2 H, q, *J* = 6.94), 3.36–3.25 (2 H, m), 1.29 (3 H, t, *J* = 7.25) ^13^C-NMR (CDCl_3_) 171.61, 166.25, 149.50, 147.77, 135.89, 133.66, 133.45, 132.66, 129.86, 129.44, 129.31, 128.61, 128.12, 127.94, 127.59, 127.21, 61.73, 53.62, 25.63, 14.16 LC/MS-MS 425.13 (M+H^+^).

**(*S*)-Ethyl 2-[2-hydroxy-5-(pyridin-3-yl)benzamido]-3-phenylpropanoate (8). **Yield 16%. mp 176 °C IR 3325, 2928, 2851, 1744, 1625, 1572, 1244 ^1^H-NMR (CDCl_3_) 12.04 (1 H, s), 8.67 (1 H, s), 8.51–8.50 (1 H, m), 7.71–7.70 (1 H, m), 7.55–7.53 (1 H, dd, *J_1_* = 8.83, *J_2_* = 2.19), 7.41–7.40 (1 H, m), 7.30–7.27 (1 H, m), 7.25–7.21 (3 H, m), 7.11–7.09 (2 H, m), 7.02 (1 H, d, *J* = 8.83), 6.97 (1 H, d, *J* = 7.25), 4.98–4.94 (1 H, m), 4.17 (2 H, q, *J* = 7.25), 3.25–3.17 (2 H, m), 1.22 (3 H, t, *J* = 7.25) ^13^C-NMR (CDCl_3_) 171.33, 169.11, 148.26, 147.82, 135.55, 133.97, 133.19, 129.35, 128.72, 127.43, 119.41, 114.56, 61.96, 33.95, 24.94, 14.14 LC/MS-MS 391.05 (M+H^+^).

**(*S*)-Ethyl 3-phenyl-2-[4-(pyrimidin-5-yl)benzamido]propanoate (9). **Yield 32%. mp 129 °C IR 3269, 2928, 1738, 1652, 1530, 1414, 1349, 1192 ^1^H-NMR (CDCl_3_) 9.24 (1 H, s), 8.97 (2 H, s), 7.88 (2 H, d, *J* = 8.51), 7.65 (2 H, d, *J* = 8.51), 7.32 (3 H, m), 7.16–7.14 (2 H, m), 6.67–6.66 (1 H, m), 5.10–5.07 (1 H, m), 4.24 (2 H, q, *J* = 7.25), 3.34–3.24 (2 H, m), 1.41 (3 H, t, *J* = 7.25) ^13^C-NMR (CDCl_3_) 171.55, 165.93, 158.06, 154.97, 137.55, 135.80, 134.42, 133.34, 129.41, 128.62, 128.16, 127.24, 61.79, 53.62, 37.90, 14.17 LC/MS-MS 376.08 (M+H^+^).

**(*S*)-Methyl 3-(1*H*-indol-3-yl)-2-[4-(pyridin-3-yl) benzamido]propanoate (10). **Yield 24%. mp 96 °C IR 3266, 1737, 1642, 1530, 1435, 1215 ^1^H-NMR (CDCl_3_) 8.75 (1 H, s), 8.55 (1 H, s), 8.40 (1 H, s), 7.80–7.77 (1 H, m), 7.70 (2 H, d, *J* = 8.51), 7.49 (3 H, s), 7.31–7.27 (2 H, m), 7.12–7.09 (1 H, m), 7.03–7.00 (1 H, m), 6.95–6.94 (1 H, m), 6.70 (1 H, d, *J* = 7.57), 5.11–5.08 (1 H, m), 3.66 (3 H, s), 3.44–3.36 (2 H, m) ^13^C-NMR (CDCl_3_) 172.38, 166.40, 149.09, 148.23, 141.04, 136.21, 134.47, 133.40, 127.94, 127.71, 127.22, 122.91, 122.31, 119.75, 118.63, 111.40, 109.95, 53.60, 52.49, 27.64 LC/MS-MS 400.08 (M+H^+^).

**(*S*)-Methyl 3-(1*H*-indol-3-yl)-2-[4-(pyrimidin-5-yl)benzamido]propanoate (11). **Yield 14%. mp 101 °C IR 3298, 1736, 1646, 1532, 1415, 1344, 1215 ^1^H-NMR (CDCl_3_) 9.23 (1 H, s), 8.95 (2 H, s), 8.26 (1 H, s), 7.81 (2 H, d, *J* = 8.20), 7.60–7.55 (3 H, m), 7.36 (1 H, d, *J* = 8.20), 7.21–7.18 (1 H, m), 7.10–7.08 (1 H, m), 7.03–7.02 (1 H, m), 6.76 (1 H, d, *J* = 7.57), 5.20–5.16 (1 H, m), 3.76 (3 H, s), 3.53–3.47 (2 H, m) ^13^C-NMR (CDCl_3_) 172.31, 166.11, 157.87, 154.94, 137.37, 136.16, 134.32, 133.38, 128.27, 127.71, 127.12, 127.00, 122.82, 122.41, 119.83, 118.62, 111.38, 109.99, 53.65, 52.55, 27.59 LC/MS-MS 400.98 (M+H^+^).

**4-(Thiophen-3-yl)benzoic acid (12). **Yield 94%. mp 280 °C (Lit. 281-282 °C) [8] ^1^H-NMR (d_6_-DMSO) 8.05–8.04 (1 H, m), 7.96 (2 H, d, *J* = 8.51), 7.85 (2 H, d, *J* = 8.20), 7.63–7.68 (1 H, m), 7.64–7.63 (1 H, m). ^13^C-NMR (d_6_-DMSO): 167.02, 140.31, 139.09, 129.92, 129.07, 127.46, 126.14, 125.99, 122.79.

**4-(Pyridin-3-yl)benzoic acid (13).** Yield 62%. mp 215 °C (Lit. 215 °C) [9] ^1^H-NMR (D_2_O/TFA) 7.80 (1 H, s), 7.61–7.56 (2 H, m), 7.00 (2 H, d, *J* = 8.51), 6.96–6.93 (1 H, m), 6.56 (2 H, d, *J* = 8.51) ^13^C-NMR was not applied due to the TFA.

**4-(Chinolin-3-yl)benzoic acid (14).** Yield 92%. ^1^H-NMR (d_6_-DMSO/TFA: 1/1) 9.66 (1 H, s), 9.51 (1 H, s), 7.80–7.78 (1 H, m), 8.38 (1 H, d, *J* = 7.88), 8.27 (1 H, d, *J* = 8.51), 8.15–8.08 (5 H, m), 7.98–7.95 (1 H, m) [Lit. 250 MHz, d_6_-DMSO: 13.09 (1 H, br s), 9.32 (1 H, d), 8.76 (1 H, d), 8.07 (5 H, m), 7.83 (2 H, m), 7.67 (1 H, m)] [10] ^13^C-NMR (d_6_-DMSO + TFA) 166.79, 144.39, 132.64, 131.15, 130.18, 127.61, 129.76, 129.47, 118.44, 116.15, 113.85, 111.56.

**5-(Pyridin-3-yl)salicylic acid (15).** Yield 46%. mp 260 °C (Lit. 263 °C) [11] ^1^H-NMR (D_2_O/TFA: 1/1) 7.61 (1 H, s), 7.42–7.40 (1 H, m), 7.36–7.35 (1 H, m), 6.93 (1 H, d, *J* = 2.52), 6.78–6.75 (1 H, m), 6.49 (1 H, dd, *J* = 8.51), 5.84 (1 H, d, *J* = 8.83) ^13^C-NMR was not applied due to the TFA.

**4-(Pyrimidin-5-yl)benzoic acid (16).** Yield 60%. mp 218 °C (Lit. 220 °C) [12] ^1^H-NMR (d_6_-DMSO) 9.17 (1 H, s), 9.15 (2 H, s), 8.01 (2 H, d, *J* = 8.20), 7.74 (2 H, d, *J* = 8.20) ^13^C-NMR (d_6_-DMSO) 169.60, 157.11, 154.60, 140.35, 133.92, 133.18, 131.47, 131.39, 129.85, 128.75, 125.81.

## Figures and Tables

**Fig. (2):**
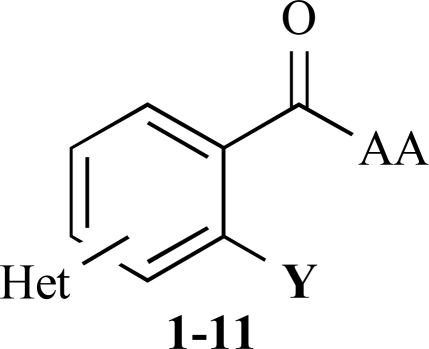
General structure of the synthesized benzoic acid amides (Het = Heterocycle, Y = -H, -OH, AA = L-alanine ethyl ester, L-phenylalanine ethyl ester, L-tryptophane methyl ester).

**Table 1:** Suzuki Coupling Reactions of Heterocycles and Boronic Acids Leading to Heterocyclic Substituted Carboxylic Acids (12-16)

Heterocycle	Boronic Acid	Product
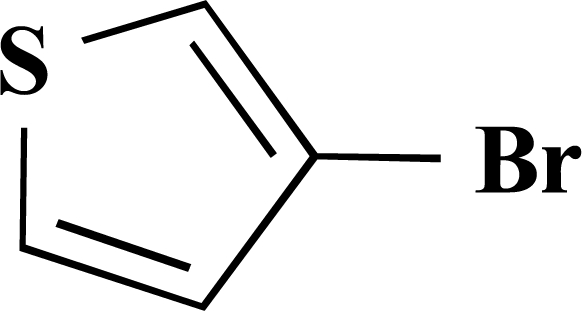	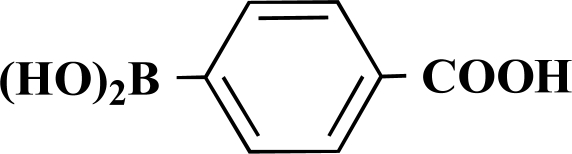	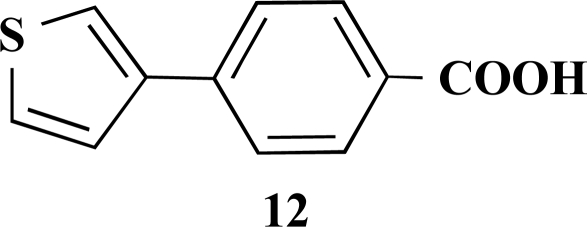
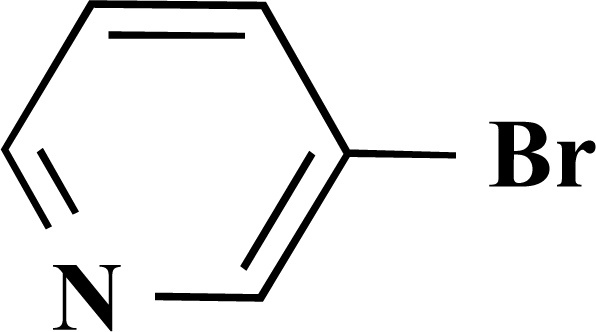	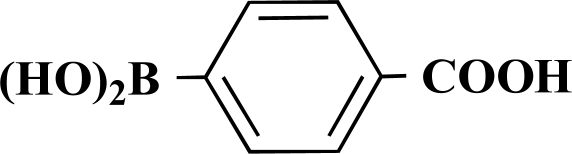	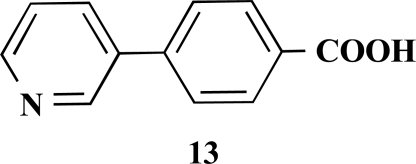
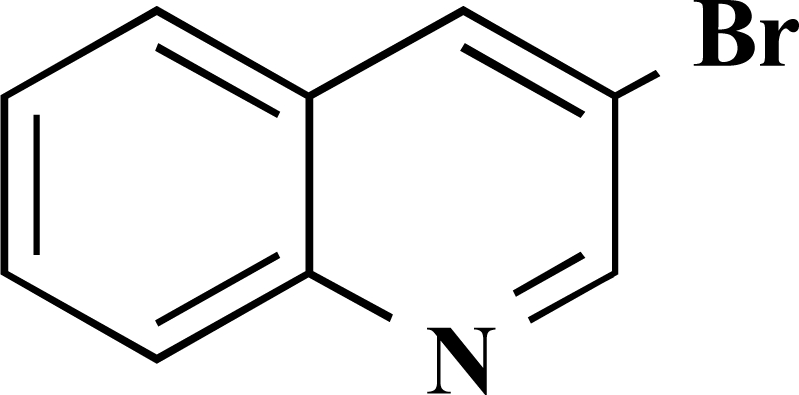	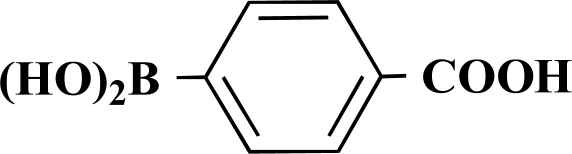	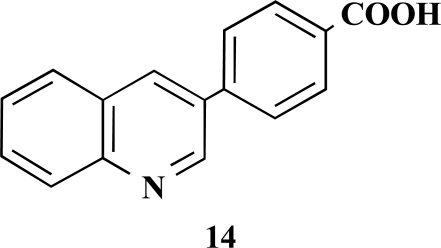
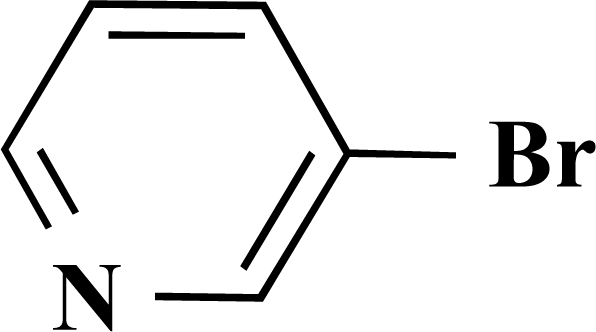	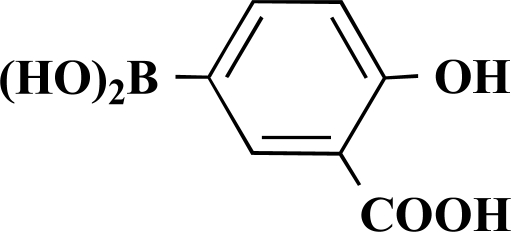	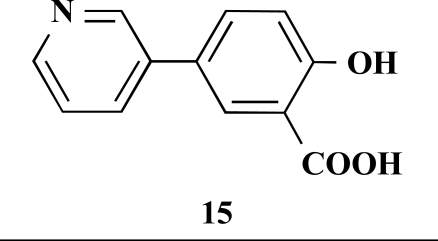
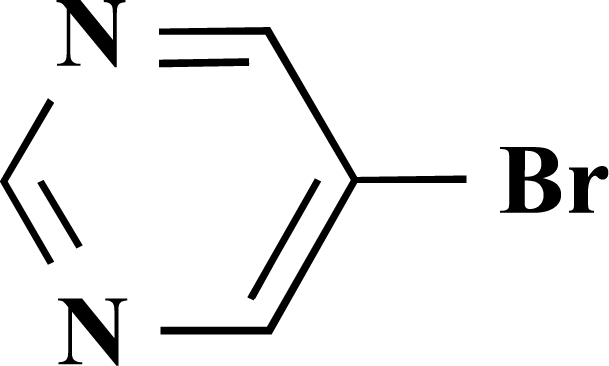	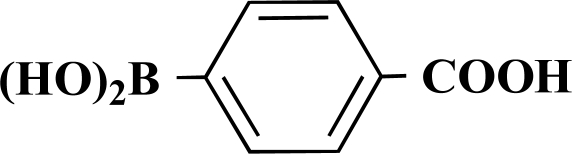	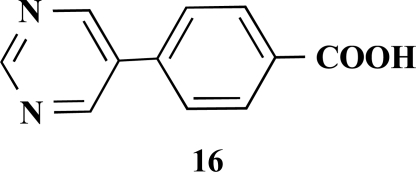

**Table 2:** Amino Acid Esters, Carboxylic Acids and the Corresponding ‘Combinatorial’ Target Compounds 1-11

	Amino Acid Ester		
Carboxylic Acids